# Association of IL1 beta gene polymorphism and allograft functions in renal transplant recipients :a case control study from Kashmir Valley

**DOI:** 10.1186/s12882-017-0526-5

**Published:** 2017-03-30

**Authors:** Mohammad Ashraf Bhat, Manzoor Ahmad Parry, Saniya Nissar, Aga Syed Sameer, Imtiyaz A. Bhat, Zafar A. Shah, Roohi Rasool

**Affiliations:** 1 Department of Nephrology, Sher-I-Kashmir Institute of Medical Sciences, Soura, Srinagar, Kashmir India; 2 Department of Immunology and Molecular Medicine, Sher-I-Kashmir Institute of Medical Sciences, Soura, Srinagar, Kashmir 190011 India; 3 Department of Basic Medical Sciences, College of Medicine, King Saud bin Abdulaziz University for Health Sciences, Jeddah, Makkah Province Saudi Arabia

**Keywords:** Interleukins, Renal transplantation, Allograft, End stage renal disease, Kashmir

## Abstract

**Background:**

Cytokines have been found to be the important mediators during renal graft outcome. Therefore, we designed this study to investigate the role of recipients’ IL-1 β promoter (−511) and IL-1 β exon-5 (+3954) polymorphisms with the risk of graft outcome.

**Methodology:**

We enrolled one hundred recipients of living-related renal transplants together with the age and sex matched controls from the healthy population not having any renal abnormality for this study. Genotype frequencies of the IL-1 β promoter (−511) and IL-1 β exon-5 (+3954) were analyzed using PCR-RFLP technique.

**Results:**

Our results revealed significant differences in the healthy control group and patient group in IL *1β +3954* (*p* < 0.001). The frequency of variant type TT genotype was higher in RE group as compared to SGF and showed 4 fold risk of rejection (OR = 4.54, *p* < 0.069) although *p* value was not significant. The frequency of wild type CC genotype and CT was not significant (*p* value 0.89 and 0.74 respectively).

**Conclusion:**

Our findings suggest that there is a prevalence of mutated allele of IL-1 gene cluster in our population, which may be responsible for renal dysfunction.

## Background

Renal Transplantation is the treatment of choice for end stage renal disease (ESRD). However, acute rejection (AR) remains important clinical problems accounting for chronic allograft loss and suboptimal long-term outcome [1, 2]. There is growing evidence of the genetic association between certain cytokine or its receptor antagonist and AR after renal transplantation. Interleukin-1 (IL-1) plays a key role in inflammatory and immune-mediated diseases. During allograft rejection, IL-1 production precedes allograft dysfunction and injury. The IL-1 system is unique in having a natural inhibitor known as the IL-1 receptor antagonist (IL-1Ra) molecule [3]. Genes for IL-1a, IL1β and IL-1Ra are located on chromosome 2q14–21 [4]. Net effect of the three members of the IL-1 gene family is to control inflammatory and host defense responses as IL-1 causes vaso-relaxation, increases adherence of lymphocytes and neutrophils to endothelial cells and might be implicated in the immune-biology of both acute and chronic graft rejection [5]. Some studies [6–8] investigated the association of IL1β, IL-1 receptor antagonist (IL-1ra) gene polymorphism with acute renal graft rejection. However, the effects of these polymorphisms on AR after renal transplantation are still controversial. It has been suggested that cytokine genotyping may play a predictive role in identifying individuals who are at higher risk of acute rejection by individualizing their immunosuppression levels [9]. We sought to ascertain whether polymorphisms of the gene encoding recipients IL-1β impacts on the incidence of acute renal graft rejection.

Our aim of study was to identify non-HLA risk factors, which may be used in pre-transplant patient assessment. Our study investigated association of IL-1 β promoter (−511) and IL-1 β exon-5 (+3954) genotypes with the risk of graft outcome. Renal transplant patients were analyzed for haplotype variation for evaluate its impact in graft outcome including rejection episode (RE) and stable graft function (SGF).

## Methods

Our study was a hospital based retrospective study that was carried out in the Departments of Internal Medicine, Nephrology and Immunology & Molecular Medicine, Sher-i-Kashmir Institute of Medical Sciences, Srinagar. Renal allograft recipients were taken in the study after informed consent and the study was ethically approved by Institutional Ethics Committee under ethical clearance no. SIMS1 131/IEC-SKIMS/2014-25 dated 30^th^ April 2013.

### Study population

Kidney Transplant recipients fulfilling the inclusion/exclusion criteria admitted in the Department of Nephrology and following Nephrology Out-Patient Department were included in this study. One hundred recipients of living-related renal transplants were enrolled in this study. Age and Sex matched controls were selected from the healthy population not having any renal abnormality.

#### Inclusion criteria

All the patients receiving Renal Allograft were taken for the study. All were live both related and unrelated with ABO compatible with maximum of 3/6 mismatch.

#### Exclusion criteria

Patients with other reasons (other than immunological cause) for transplant rejection or failure like Technical Problems, Graft failure due to any drug(like NSAIDS, herbal medications and other nephrotoxic drugs),or dye intake, uro-obstruction, infection, surgical failure, recurrence of original disease in transplanted kidney, Cyclosporine(CsA)/Tacrolimus toxicity etc. Patient who died with a functioning graft during the study period was not included in the study.

All patients received an immunosuppressive regimen consisting of steroids, Tacrolimus and mycophenolate mofetil. The steroid regimen consisted of intravenously administered methylprednisolone 500 mg at the time of surgery followed by intravenously administered methylprednisolone 1 g/d for the next 3 days, at which time the medication regimen was changed to oral prednisolone 30 mg/d, which was progressively tapered to 15 to 20 mg/d by the end of the first postsurgical month. An increase in the serum creatinine level that was > = 10% from the baseline value and that could not be attributed to a cause such as urinary flow obstruction, a toxic reaction to cyclosporine, or a urinary tract infection was defined as clinical rejection. Episodes of acute clinical rejection were recorded and treated with steroid pulse therapy. If the patient had no clinical response to that treatment, a kidney biopsy was performed. Biopsy specimens were graded according to the 2009 Banff criteria.

### Methodology

**Fig. 1:**
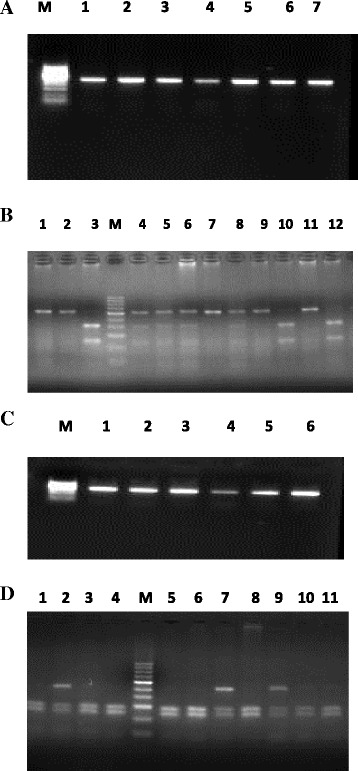
**a** Representative PCR amplification picture of *IL-1B* -51 T/C. Lanes 1–7: 304 bp amplified PCR product of *IL-1β* -51 T/C, Lane M: 50 bp DNA Marker. **b** RFLP picture of *IL-1β* after restriction digestion with Ava1 1 (3%) agarose gel electrophoresis. Lane M: 50 bp marker, Lane 1, 2, 7, 9, And 11: homozygous wild TT genotype, Lane 4, 5, 6, 8: heterozygous TC genotype, Lane 3,10,12 : Homozygous variant CC genotype. **c** Representative PCR amplification picture of *IL-1β +3954.* Lanes 1–6:249 bp amplified PCR product of *IL-1β + 3954,* Lane M: 50 bp DNA Marker. **d** RFLP picture of *IL-1β* after restriction digestion with Taq 1 (3%) agarose gel electrophoresis. Lane M: 50 bp marker, Lane 1,3,4,5,6,8,10,11: homozygous wild CC genotype, Lane 2,7,9: heterozygous TC genotype

- Peripheral blood of the patient was collected in 5 ml EDTA vial after transplantation and was stored at −80 °C till further use.
- DNA extraction was done by Phenol/Chloroform method or DNA extraction kit.
- Polymerase Chain Reaction Amplification of the desired region was done using specific primers and followed by Restriction Fragment Length Polymorphism method by Enzyme digestion method.
- Digestion products were checked on 3% Agarose gel by electrophoresis.
- Patients were followed for detection of rejection of graft function or graft dysfunction for minimum 1 year [See Fig. 1 for details].

### Statistical analysis

Data was analyzed with the help of percentage statistics. Allele and genotype frequencies were compared through a 2x2 contingency table using chi-square test or Fischer’s exact test, whichever appropriate. Odds ratio (OR) with 95% confidence interval (CI) was determined for the disease susceptibility of patients. Logistic regression analysis was performed to investigate the impact of other prognostic factors like age, gender with graft outcome. Statistical analysis was performed with help of SPSS software version 16. *P*-values less than 0.05 were considered statistically significant.

## Results

### Association of subject characteristics

Allograft function association with characteristics like age and gender was seen by logistic regression studies which showed no significant association as shown in Table 1.

**Table 1:** Association of subject characteristics

Characteristic	*SGF (n = 61)*	*Rejection Episodes (n = 30)*
Age (Yrs)	*16–63*	*18–60*
Sex		
Male	*46 (75.4%)*	25 (83.3%)
Female	*15 (24.6%)*	5 (16.7%)
*Donor Age*		
*< 50*	47 (77.1%)	*24 (80%)*
*> 50*	14 (22.9%)	*6 (20%)*
*CGN*	*51*	*28*
Othersª	*10*	*2*

*CGN* chronic glomerulonephritis

^a^ *others include Hypertension¸Type 2 DM, Vasculitis, RPGN* rapidly proliferative glomerulonephritis

### IL 1 genotypes and outcome of allograft function

Genotype frequency distributions for IL 1β exon-5(+3954) among renal transplant patients are shown in Table 2. The frequency distribution was in Hardy-Weinberg equilibrium. The results revealed significant differences in the healthy control group and patient group in IL *1β +3954* (*p* < 0.001). The frequency of C/C was higher in patients as compared to controls (41.8 vs 16%). No significant difference was observed between RE and SGF. The frequency of T/T genotype was higher in RE group as compared to SGF and showed 4 fold risk of rejection (OR = 4.54, *p* < 0.069) although *p* value was not significant. The frequency of C/C genotype and C/T was not significant (*p* value 0.89 and 0.74 respectively).

**Table 2:** *IL 1β +3954* T/C genotypes frequencies in transplant recipients as a whole and grouped and controls

Genotypes	CC (%)	CT (%)	TT (%)	No. Of Alleles C/T
Patients (*n* = 91)	38 (41.8%)	47 (51.6%)	06 (6.6%)	123/59 (67.6/32.4)
Controls (*n* = 200)	32 (16%)	110 (55%)	58 (29%)	174/226 (43.5/56.5)
OR at 95% CI	3.76 (2.144–6.608)	0.874 (0.532–1.436)	0.173 (0.071–0.418)	2.71 (1.874–3.913)
RE (*n* = 30)	12 (40%)	14 (46.7%)	4 (13.3%)	36/22 (62.1/37.9)
SGF (*n* = 61)	26 (42.6%)	33 (54.1%)	2 (3.3%)	85/37 (69.7/30.3)
*P* – value and OR at 95% CI (RE vs SGF)	0.811; OR = 0.89 (0.369–2.185)	0.505; OR = 0.74 (0.309–1.784)	0.069; OR = 4.54 (0.781–26.36)	0.309; OR = 0.71 (0.369–1.373)
Steroid responsive RE (*n* = 24)	9 (37.5%)	11 (45.83%)	4 (16.66%)	29/19 (60.4/39.6)
Steroid Resistant RE (*n* = 6)	3 (50%)	3 (50%)	0	9/3 (75/25)

Genotype frequency distributions for IL 1β promoter (−511) among renal transplant patients are shown in Table 3. The results revealed significant differences between the healthy control and the patient groups in IL-1β promoter region − 511 (*p* < 0.001). The frequency of E1/E1 genotype was higher in patients as compared to controls (45.1 vs.29%) and showed more than 2 folds increased risk in patients(OR = 2.01). The frequency of E1/E1 was also higher in RE group as compared to SGF (60 vs. 36.1%) and showed 2.6 fold risk for RE (OR = 2.66, *p* = 0.03)

**Table 3:** Genotype and allele frequencies of IL 1β -511 in transplant recipients as a whole and grouped and controls

Genotypes	E1/E1 (%)	E1/E2 (%)	E2/E2 (%)	No. Of Alleles C/T
Patients (*n* = 91)	41 (45.1%)	45 (49.4%)	5 (5.5%)	55/127 (30.2/69.8)
Controls (*n* = 200)	58 (29%)	110 (55%)	32 (16%)	174/226 (43.5/56.5)
OR at 95% CI	2.01 (1.201–3.356)	0.800 (0.487–1.315)	0.305 (0.115–0.812)	0.563 (0.387–0.817)
RE (*n* = 30)	18 (60%)	11 (36.7%)	01 (3.3%)	13/47 (21.7/78.3)
SGF (*n* = 61)	22 (36.1%)	35 (57.4%)	4 (6.5%)	43/79 (35.2/64.8)
*P* – value and OR at 95% CI (RE vs SGF)	0.031; OR = 2.66 (1.083–6.530)	0.063; OR = 0.43 (0.175–1.057)	0.885; OR = 0.491 (0.052–4.602)	0.062; OR = 0.508 (0.248–1.042)
Steroid responsive RE (*n* = 24)	16 (66.6%)	8 (33.33%)	0	40/8 (83.3/16.6)
Steroid Resistant RE (*n* = 6)	3 (50%)	2 (33.33%)	1 (16.66)	8/4 (66.66/33.3)

### Distribution of haplotypes and linkage disequilibrium

Haplotype frequencies among the two IL-1 gene polymorphisms in patients and controls and RE and SGF are shown in Table 4. There was a significant difference in haplotype C-E1 frequency distribution among patients and controls (OR = 1.557 *p* value = 0.016). All the four haplotypes were present in rejection episodes and stable graft function. There was non-significant difference between Rejection episodes and stable graft function.

**Table 4:** Association of patient IL-1ß (promoter region and exon-5 in rejecters (RE) and non-rejecters (SGF)

No.	Haplotype	Patient (%)	Control (%)	*p*-value	OR	RE (%)	SGF (%)	*p*-value	OR(RE vs. SGF)
1	C/E1	47 (23.3)	87 (21.8)	0.673	1.091	10 (16.7)	37 (26.1)	0.149	0.568
2	C/E2	75 (37.1)	110 (27.5)	0.016	1.557	25 (41.7)	50 (35.2)	0.568	0.386
3	T/E1	33 (16.3)	90 (22.5)	0.077	0.673	8 (13.3)	25 (18.6)	0.453	0.72
4	T/E2	47 (23.3)	113 (28.2)	0.191	0.77	17 (28.3)	30 (21.1)	0.268	1.476

## Discussion

Despite advances in immunosuppressive therapy, acute rejection remains an important cause of transplant injury. For the past decade, the influence of cytokine gene polymorphisms has been studied in patients with autoimmune disorders and those who have undergone solid-organ transplantations, and the results of those investigations are inconsistent. IL-1 plays a key role in inflammatory and immune-mediated diseases. During allograft rejection, IL-1 production precedes allograft dysfunction and injury. Marked inter-individual and intra-individual variations in the production of IL-1β, IL-10 TNF-α, IL-2, IL-4, IFN-γ, and other cytokines have been reported, but attempts to define the effects of particular genotypes on cytokine production, both in vitro and in vivo, have produced inconsistent results.

In our study a total of 91 patients of allograft renal transplant were taken who have undergone transplant after proper immunologic evaluation including cytotoxic cross matching, HLA typing and ABO typing. 30 patients that constitute 32% developed rejection episodes over a period of 2 years. 24(80%) patients out of these rejection episodes responded to steroids, while as 6(20%) patients were steroid resistant.

IL1β 511(C/T) polymorphism has been studied in various studies regarding allograft function. We found significant difference in the genotypic distribution of IL-1 gene cluster among patients and controls. The IL 1β (+*3954)* CC genotype was observed more frequently in the patients (41.8%) than the controls (16%) (OR = 3.76). The IL 1β (−*511)* TT genotype was observed more frequently in the patients (45.1%) than the controls (29%) (OR = 2.01). These results were consistent with the studies of various other studies [6, 7, 10, 11]. This suggests that there is prevalence of mutated allele of IL-1 gene cluster in our population, which is responsible for allograft rejection.

Manchanda et al., studied polymorphism in relation with rejection episodes and stable graft function. They observed that there was a non-Significant association between IL-1 (promoter region −511) and chronic rejection among kidney recipients, which was consistent with the results of Uboldi de Capei et al [11, 12]. Seyhun et al. observed no significant association of IL 1β polymorphism with rejection episodes as was seen by Jie Zhao et al. in 2013 in Chinese population [13]. We observed T/T genotype of IL 1β promoter −511 had >2.6 risk of rejection (OR = 2.66 *p* =0.031) which was significant and consistent with results of Manchanda. It may be due to the fact that allele 2 of IL1-511 (E1) is assumed to represent a “high secretor” phenotype leading to increased pro-inflammatory activity in autoimmune and infectious diseases [14]. The frequency distribution of IL 1β exon (+3954) varied among rejection episodes and stable graft function. The T/T genotype showed 4 fold risk of rejection although it had low statistical power (OR = 4.54 *p* = 0.069). Our results were consistent with Manchanda who showed 2 fold risk of rejection. It has been previously reported that presence of genotype T/T of IL-1 exon-5 + 3954 increases pro-inflammatory activity of IL-1 [15]. Thus, overproduction of IL-1 during kidney transplant rejection may promote allograft dysfunction and injury.

Earlier studies suggested the importance of IL-1 haplotype reflecting differential regulation of IL-1Ra expression by IL-1β and coordinated effects of polymorphisms that regulate IL-1 bioactivity in vivo [16]. Manchanda observed that there was significant difference IL-1β promoter, exon-5 haplotype distribution between RE and SGF. We constructed haplotypes for IL 1β polymorphism in our study. There was a significant difference in haplotype C-E1 frequency distribution among patients and controls (OR = 1.557 *p* value = 0.016). IL 1β gene (promoter −511 and exon +3954) haplotype frequencies were comparable in RE, SGF. Before embarking on large-scale haplotype-based studies on samples from genetically distinct populations, it is important to consider variation both in linkage disequilibrium (LD) and in haplotype frequencies within and across populations, as both vary considerably. Previous functional studies suggest that the combination of alleles may be an important aspect in the regulation of IL-1 gene expression [17].

## Conclusion

Our study suggests that there is prevalence of mutated allele of IL-1 gene cluster in our population, which may be responsible for allograft rejection.

## Abbrevations

AR: Acute rejection
CsA: Cyclosporine
ESRD: End stage renal disease
IL1 Ra: Interleukin 1 receptor antagonist
IL1: Interleukin 1
RE: Rejection episode
SGF: Stable graft function
β: beta
